# Gaming Disorder: looking for a specific psychopathological profile in a Russian sample

**DOI:** 10.1192/j.eurpsy.2022.354

**Published:** 2022-09-01

**Authors:** D. Dovbysh, N. Bogacheva, V. Epishin

**Affiliations:** Federal State Autonomous Educational Institution of Higher Education I.M. Sechenov First Moscow State Medical University under the Ministry of Health of the Russian Federation (Sechenov University), Pedagogy And Clinical Psychology, Moscow, Russian Federation

**Keywords:** Gaming Disorder

## Abstract

**Introduction:**

ICD-11 describes Gaming disorder as a behavioral pattern characterized by impaired control over gaming, increased gaming priority, and escalation despite consequences. This description is similar to other addictive behaviors with minor specifics. However, it is unclear if gaming disorder has any specific psychopathological profile.

**Objectives:**

The study aimed to investigate gaming disorder’s connection to primary psychopathological symptomatology.

**Methods:**

515 gamers aged 16-56 (75% male) anonymously completed online questionnaires: SCL-90-R and Video Games Addiction Scale (VGAS) – our new 26-items questionnaire based on ICD-11 criteria for gaming disorder. VGAS showed good reliability (Cronbach`s α=0.858) and external validity (positive correlation with Chen Internet Addiction Scale, r=0.472, p=0.000).

**Results:**

Gaming disorder severity showed positive correlations (p=0.000) with all SCL-90-R scales. Pearson`s r ranged from 0.311 (phobic anxiety) to 0.431 (depression). Thus, gaming disorder showed no specific combination of psychopathological symptoms. Instead, all symptoms had almost equal correlations with the VGAS score.

**Conclusions:**

Gaming disorder is not linked to any particular combination of psychopathological symptoms. On the contrary, as suggested by our study, different symptoms are almost equally related to excessive gaming. Several interpretations are possible. Problematic gaming can be a way for psychologically distressed people to deal with different symptoms. Conversely, gaming disorder can itself lead to psychological maladjustment. Thus, further thorough research is required, specifically when deciding on the primary diagnosis in comorbid cases or choosing the therapeutic aims.

**Disclosure:**

No significant relationships.

